# Real-world effectiveness of burosumab across age groups: X-linked hypophosphatemia (XLH) Disease Monitoring Program

**DOI:** 10.1210/clinem/dgag140

**Published:** 2026-04-04

**Authors:** Leanne M Ward, Thomas O Carpenter, Hamilton Cassinelli, Pablo Florenzano, Erik A Imel, Aliya A Khan, Ben Johnson, Erru Yang, Marc Vincent, Heather M Heerssen, Zhiyi Li, Jill H Simmons

**Affiliations:** Children's Hospital of Eastern Ontario, University of Ottawa, Ottawa, ON, Canada K1H 8L1; Departments of Pediatric (Endocrinology), and Orthopedics and Rehabilitation, Yale University School of Medicine, New Haven, CT 06510, USA; Department of Endocrinology, Ricardo Gutiérrez Children's Hospital, C1425 Buenos Aires, Argentina; School of Medicine, Pontificia Universidad Católica de Chile, 8331150 Santiago, Chile; Departments of Medicine and Pediatrics, Indiana University School of Medicine, Indianapolis, IN 46202-3082, USA; Division of Endocrinology and Metabolism, McMaster University, Hamilton, ON, Canada L8S 4L8; Kyowa Kirin International plc, Marlow, Buckinghamshire, SL7 1HZ, UK; Ultragenyx Pharmaceutical Inc., Brisbane, CA 94005, USA; Kyowa Kirin Inc., Princeton, NJ 08540, USA; Kyowa Kirin Inc., Princeton, NJ 08540, USA; Kyowa Kirin Inc., Princeton, NJ 08540, USA; School of Medicine, Vanderbilt University, Nashville, TN 37232, USA

**Keywords:** burosumab, patient-reported outcomes, phosphate, real-world, X-linked hypophosphatemia

## Abstract

**Context:**

X-linked hypophosphatemia (XLH) is a rare disorder characterized by excess fibroblast growth factor 23 (FGF23), leading to chronic hypophosphatemia, osteomalacia, and rickets.

**Objective:**

To evaluate outcomes up to 3 years following initiation of treatment with anti-FGF23 antibody (burosumab) in a real-world setting among individuals with XLH, stratified by age group, including those excluded from clinical trials (<1, 13-17, and ≥65 years).

**Methods:**

The XLH Disease Monitoring Program is a prospective, longitudinal, long-term-outcomes program for individuals with XLH. This analysis included participants who were burosumab-naive at baseline and who had initiated burosumab between baseline and the Year 1 visit. Changes from baseline in biochemistry, clinical outcomes, and patient-reported outcomes (PROs) were assessed at Year 1 (Y1) and Year 3 (Y3) visits.

**Results:**

Among participants (*n* = 139), burosumab led to significant and sustained improvements in mean (SD) serum phosphate *z*-scores at Y1 and Y3 (change from baseline: 1.4 [1.1]; *P* < .0001 for each timepoint). Among pediatric participants, serum alkaline phosphatase z-scores, Rickets Severity Scores, and patient-reported pain interference declined significantly at Y1 and Y3; non-significant changes were observed in fatigue and physical function mobility. Adults showed significant improvements in PRO measures of pain, stiffness, and physical function, with higher proportions of participants achieving minimal clinically important differences at Y3 vs Y1. Trends observed in overall cohorts were generally consistent across age sub-groups, with statistical significance reached for many endpoints.

**Conclusion:**

This analysis demonstrated the real-world effectiveness of burosumab for XLH, which appears evident across age groups.

X-linked hypophosphatemia (XLH) is a rare disorder caused by pathogenic variants in the phosphate-regulating endopeptidase homolog X-linked (*PHEX*) gene (1, 2). These genetic variants lead to excess production of fibroblast growth factor 23 (FGF23), resulting in chronic renal phosphate wasting and hypophosphatemia (3). In children with XLH, chronic hypophosphatemia causes osteomalacia and rickets, which are characterized by deficient bone and cartilage mineralization, delayed endochondral ossification, slowing of growth velocity, short stature, and persistent lower limb deformity (4, 5). Children and adults with XLH experience dental issues including caries and abscesses (6, 7). Adults with XLH also experience osteomalacia, which is associated with bone pain and increased risk of pseudofractures. The adult XLH phenotype is further characterized by progressive musculoskeletal morbidity including stiffness, osteoarthritis, enthesopathy, osteophytes, and syndesmophytes (8-10).

Treatment for XLH over the past 40 years has consisted of multiple daily doses of phosphate salts combined with one or more daily doses of active vitamin D (Pi/D); however, treatment results can be variable, and careful monitoring is needed to avoid adverse effects of Pi/D therapy, including nephrocalcinosis, hypercalciuria, hyperparathyroidism, and gastrointestinal side effects (1).

Burosumab is a fully human monoclonal antibody that binds to FGF23 and inhibits its activity. In April 2018, burosumab was approved by the US Food and Drug Administration (FDA) for the treatment of patients with XLH aged ≥ 1 year in the United States, followed by region-specific approvals in various parts of the world. In September 2019, FDA approval was expanded to include patients aged 6-12 months (11).

The efficacy and safety of burosumab have been demonstrated in pivotal phase 3 clinical trials in both pediatric and adult participants with XLH (12, 13). Children aged 1-12 years who received burosumab at a dose of 0.8-1.2 mg/kg subcutaneously (SC) every 2 weeks for 64 weeks showed significant improvements in alkaline phosphatase activity at 16, 40, and 64 weeks, serum phosphate concentration and rickets severity at 40 and 64 weeks, and mobility at 64 weeks, compared with those who received Pi/D (12).

Among adults with XLH, the safety and efficacy of burosumab were evaluated in a 24-week, double-blind, placebo-controlled phase 3 clinical trial. Participants who received burosumab 1 mg/kg SC every 4 weeks showed significant improvements in serum phosphate concentration and reduced stiffness scores as measured using the Western Ontario and McMaster Universities Osteoarthritis Index (WOMAC), and improved healing of fractures and pseudofractures compared with placebo (13). In a subsequent open-label extension of the same study, adults treated with burosumab achieved meaningful and significant improvements from baseline in pain and all WOMAC domains by Week 48. Furthermore, meaningful and statistically significant improvements in ambulatory function were observed by Week 96 (14, 15).

The purpose of the current analysis was to evaluate the real-world effectiveness of burosumab over a longer duration (up to 3 years) across a broad range of age groups, including individuals with XLH aged < 1 year, 13-17 years, and ≥ 65 years who were excluded from the above-mentioned clinical trials (12-15).

## Materials and methods

### Study design and patients

The XLH Disease Monitoring Program (XLH-DMP; NCT03651505) is a multinational, multicenter, longitudinal, long-term, prospective, observational initiative designed to characterize disease presentation and progression of XLH and assess the long-term safety and effectiveness of burosumab treatment. The study was approved by each site's Institutional Review Board or Ethics Committee. Data collection began on July 16, 2018, and is intended to continue until December 2032.

Participants were eligible to be enrolled in the XLH-DMP if they had a confirmed diagnosis of XLH based on characteristic clinical features and a biochemical profile consistent with XLH or if they or an affected family member had a confirmed pathogenic or likely pathogenic *PHEX* variant. Enrollment eligibility was independent of treatment status and included participants receiving burosumab, Pi/D, or no medical therapy. Participants were permitted to enroll in the XLH-DMP irrespective of any planned or unplanned changes to their XLH-specific treatment regimen during the 10-year course of the study. People were excluded from the XLH-DMP if they had serious medical or psychiatric comorbidities, or the study investigator assessed their life expectancy to be <1 year. All participants or their caregivers (the latter, if considered minors) provided informed consent. Minors provided assent when able based on local regulations.

**Figure 1:**
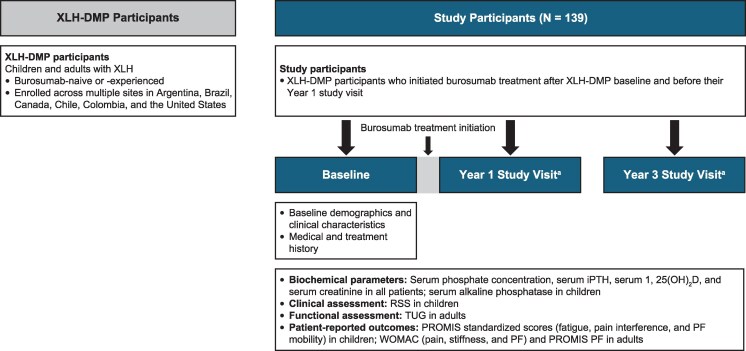
Study design. ^a^Due to the real-world nature of the study, the duration from DMP baseline to the Year 1 visit and Year 3 visit may not be exactly 1 year or 3 years and may differ across patients. Abbreviations: 1,25(OH)_2_D, 1,25-dihydroxyvitamin D; DMP, Disease Monitoring Program; iPTH, intact parathyroid hormone; PF, physical function; PROMIS, Patient-Reported Outcomes Measurement Information System; RSS, Rickets Severity Score; TUG, Timed Up and Go; WOMAC, Western Ontario and McMaster Universities Osteoarthritis Index; XLH, X-linked hypophosphatemia.

The current analysis includes participants who initiated burosumab treatment after XLH-DMP enrollment (ie, after baseline) and before their Year 1 visit, to evaluate burosumab treatment response with sufficient follow-up (up to 3 years post-burosumab initiation) (Fig. 1). Post hoc analyses were performed on sub-groups of participants stratified by age (<1, 1-4, 5-12, 13-17, 18-29, 30-39, 40-49, 50-64, and ≥ 65 years) and the total cohort, based on data that were collected prospectively for the outcomes predefined in the DMP protocol. The data reported in this analysis had a database lock date of February 24, 2024.

### Assessment of treatment duration

Treatment duration was calculated as the interval between the first dose date and the Year 1 and Year 3 visit dates. The interval between the last dose prior to the Year 1 visit and the Year 3 visit date was also assessed. As the XLH-DMP program is a real-world study, Year 1 and Year 3 visits could occur on any day within the burosumab dosing cycle.

### Study assessments

Demographic information, disease and clinical characteristics, and medical and treatment history were assessed at baseline. Biochemical parameters, rickets severity, physical performance (Timed Up and Go [TUG] test, adult only), and PROs were assessed at baseline, Year 1, and Year 3 visits (Fig. 1).

Biochemical assessments were performed per protocol after fasting (minimum 4 hours) and analyzed centrally by LabCorp. These included serum phosphate concentration (mg/dL), serum alkaline phosphatase (U/L, in children only), serum intact parathyroid hormone (iPTH) (pg/mL), serum 1,25-dihydroxyvitamin D [1,25(OH)_2_D] (pg/mL), and serum creatinine (mg/dL). As serum phosphate and alkaline phosphatase naturally decline with age, these parameters were converted to z-scores relative to age- and sex-matched reference populations (16-18).

Rickets severity in children was assessed using the Thacher Rickets Severity Score (RSS), a validated scoring system based on radiographic images of the wrists and the knees. Each site is scored separately (wrist: 0-4; knee: 0-6), with the total score (0-10) representing the sum of the 2, where higher scores indicate greater severity. The RSS provides a standardized method to evaluate skeletal manifestations of rickets and monitor treatment response over time (19).

Physical performance was assessed in adults using the TUG test, a widely used clinical assessment of functional mobility, balance, and fall risk. This test measures the time taken for an adult to rise from a chair, walk 3 meters, turn, return, and sit down, with longer times indicating greater impairment (20).

PROs were assessed in adults using the WOMAC index, which consists of 24 items in total: 5 items contribute to the pain subscale, 2 items to the stiffness subscale, and 17 items to the physical function (PF) subscale. The total score includes responses to all WOMAC items. Scoring is a summed metric normalized to 0-100, representing the percent of the maximum score, where 0 is the best state of health and 100 is the worst (21).

PROs were also assessed using Patient-Reported Outcomes Measurement Information System (PROMIS) scores. PROMIS measures use a T-score metric in which 50 is the mean and 10 is the SD of a reference population (representative of the 2000 United States General Census) (22). Higher scores indicate more of the concept being measured (ie, more fatigue, more pain interference, more mobility, or greater physical function) (22). Adult participants completed a PROMIS PF custom form, comprising 9 items, to assess self-reported physical capabilities, including mobility, upper extremity function, and the ability to perform daily activities (22, 23). In children, PROMIS scores for fatigue, pain interference, and PF mobility were assessed using age-appropriate custom forms: a self-report/parent-proxy form in children aged 8-17 years and a parent/caregiver report in children aged 5-7 years. The fatigue subscale comprises 10 items, the pain interference subscale 8 items, and the PF mobility subscale 7 items.

### Safety

Safety data were collected across the entire XLH-DMP cohort. The main safety objectives for the XLH-DMP were to evaluate the risk of nephrocalcinosis (assessed by ultrasound and a single central reader with grading on a 5-point scale) (24), renal failure, and spinal stenosis in participants treated with burosumab. Additional data were collected on pregnancies, live births, and adverse events (AEs) during pregnancy and/or lactation.

### Statistical analysis

Continuous variables were summarized as non-missing observations (*n*), mean, SD, median, interquartile range (IQR), and range. Categorical variables were summarized as count and proportion in each category evaluated. Missing values were noted and treated as missing with no imputation. For subgroups large enough to test, data distribution was generally suitable for parametric analysis (a Shapiro–Wilk test was used to confirm that continuous outcomes are approximately normally distributed in most cases); therefore, changes in outcomes from baseline to Year 1 and from baseline to Year 3 visits were compared with 0 (ie, no change) using single-sample two-tailed *t*-tests at the 5% significance level. For all biochemical parameters, the proportion of participants within the reference range (provided by the central laboratory for the DMP study, reported in Table S1) was assessed at the Year 1 and 3 study visits (25, 26). For WOMAC scores, the proportion of participants experiencing changes greater than the XLH-specific minimal clinically important differences (MCIDs) at Year 1 and Year 3 (pain > 11, stiffness > 10, PF > 8) was calculated (14). As this is an observational study, no adjustments for multiple comparisons were made; all *P*-values were considered nominal.

## Results

### Participant demographics and medical and treatment history

Among the overall cohort of 139 participants, 99/139 (71.2%) were female with a mean (SD) age of 24.5 years (18.5) at baseline. Most of the cohort were White (97/139 [69.8%]) and from the United States (89/139 [64.0%]) (Table 1). *PHEX* gene variants were confirmed in 47/66 pediatric participants and 34/73 adult participants. Among the overall cohort, the most frequently reported musculoskeletal abnormality was genu varum in 109/139 participants (78.4%), followed by intoeing in 61/139 participants (43.9%), enthesopathy/bone spurs/osteophytes in 37/139 participants (26.6%), and osteoarthritis in 32/139 participants (23.0%). The prevalence of enthesopathy/bone spurs/osteophytes, osteoarthritis, tinnitus, and hearing loss generally increased with age (Table 2).

**Table 1:** **Baseline characteristics**.

Characteristic	< 1 year (*n* = 1)	1-4 years (*n* = 22)	5-12 years (*n* = 29)	13-17 years (*n* = 14)	18-29 years (*n* = 17)	30-39 years (*n* = 24)	40-49 years (*n* = 17)	50-64 years (*n* = 12)	≥65 years (*n* = 3)	All pediatric participants (*n* = 66)	All adult participants (*n* = 73)	All participants (n = 139)
Female sex, *n* (%)	0 (0.0)	12 (54.6)	18 (62.1)	13 (92.9)	12 (70.6)	20 (83.3)	14 (82.4)	9 (75.0)	1 (33.3)	43 (65.2)	56 (76.7)	99 (71.2)
Age, years												
Mean (SD)	0.8 (0.0)	2.7 (1.1)	9.2 (2.2)	15.6 (1.6)	22.7 (3.6)	35.4 (3.0)	44.3 (3.5)	56.0 (4.0)	68.1 (2.4)	8.2 (5.2)	39.2 (13.0)	24.5 (18.5)
Median (Q1, Q3)	0.8 (0.8, 0.8)	2.7 (1.7, 3.3)	9.1 (7.9, 11.1)	15.1 (14.4, 16.7)	21.7 (19.3, 26.5)	35.3 (33.1, 38.3)	43.2 (41.0, 48.0)	55.6 (54.0, 57.1)	67.3 (66.3, 70.8)	8.1 (3.3, 11.9)	38.7 (30.6, 48.2)	19.1 (8.1, 39.3)
Min-max	0.8-0.8	1.0-5.0	5.0-12.8	13.1-18.0	18.7-29.2	30.4-39.5	40.2-49.7	50.2-65.0	66.3-70.8	0.8-18.0	18.7-70.8	0.8-70.8
Race, *n* (%)												
White	1 (100.0)	14 (63.6)	17 (58.6)	9 (64.3)	15 (88.2)	19 (79.2)	14 (82.4)	5 (41.7)	3 (100.0)	41 (62.1)	56 (76.7)	97 (69.8)
Non-white	0 (0.0)	1 (4.6)	7 (24.1)	3 (21.4)	1 (5.9)	0 (0.0)	1 (5.9)	2 (16.7)	0 (0.0)	11 (16.7)	4 (5.5)	15 (10.8)
Unknown/not reported	0 (0.0)	7 (31.8)	5 (17.2)	2 (14.3)	1 (5.9)	5 (20.8)	2 (11.8)	5 (41.7)	0 (0.0)	14 (21.2)	13 (17.8)	27(19.4)
Ethnicity, *n* (%)												
Hispanic/Latino	0 (0.0)	8 (36.4)	10 (34.5)	4 (28.6)	2 (11.8)	2 (8.3)	4 (23.5)	2 (16.7)	0 (0.0)	22 (33.3)	10 (13.7)	32 (23.0)
No Hispanic/Latino	1 (100.0)	7 (31.8)	16 (55.2)	9 (64.3)	14 (82.4)	17 (70.8)	12 (70.6)	6 (50.0)	3 (100.0)	33 (50.0)	52 (71.2)	85 (61.2)
Other	0 (0.0)	7 (31.8)	3 (10.3)	2 (7.1)	1 (5.9)	5 (20.8)	1 (5.9)	4 (33.3)	0 (0.0)	11 (16.7)	11 (15.1)	22 (15.8)
Country, *n* (%)												
Argentina	0 (0.0)	4 (18.2)	4 (13.8)	2 (14.3)	1 (5.9)	1 (4.2)	1 (5.9)	0 (0.0)	0 (0.0)	10 (15.2)	3 (4.1)	13 (9.4)
Brazil	0 (0.0)	1 (4.6)	2 (6.9)	1 (7.1)	0 (0.0)	1 (4.2)	2 (11.8)	0 (0.0)	0 (0.0)	4 (6.1)	3 (4.1)	7 (5.0)
Canada	0 (0.0)	7 (31.8)	6 (20.7)	2 (14.3)	3 (17.7)	4 (16.7)	1 (5.9)	5 (41.7)	0 (0.0)	15 (22.7)	13 (17.8)	28 (20.1)
Colombia	0 (0.0)	0 (0.0)	1 (3.5)	0 (0.0)	0 (0.0)	0 (0.0)	0 (0.0)	1 (8.3)	0 (0.0)	1 (1.5)	1 (1.4)	2 (1.4)
USA	1 (100.0)	10 (45.5)	16 (55.2)	9 (64.3)	13 (76.5)	18 (75.0)	13 (76.5)	6 (50.0)	3 (100.0)	36 (54.6)	53 (72.6)	89 (64.0)
Height *z*-score*^a^*												
*n*	1	22	29	14	17	24	14	12	3	66	70	136
Mean (SD)	−7.4 (0.0)	−2.5 (1.9)	−1.8 (1.3)	−2.7 (1.3)	−1.0 (0.6)	−0.8 (0.6)	−0.9 (0.8)	−1.3 (0.8)	−1.1 (0.5)	−2.3 (1.7)	−1.0 (0.7)	−1.6 (1.4)
Median (Q1, Q3)	−7.4 (−7.4, −7.4)	−2.7 (−4.1, −1.5)	−1.8 (−3.2, −0.8)	−2.7 (−3.9, −1.5)	−1.1 (−1.4, −0.5)	−0.8 (−1.2, −0.5)	−0.9 (−1.4, −0.5)	−1.5 (−1.7, −0.6)	−1.3 (−1.5, −0.6)	−2.4 (−3.3, −1.2)	−0.9 (−1.5, −0.5)	−1.3 (−2.5, −0.7)
Min-max	−7.4-−7.4	−5.46-2.75	−3.7-0.4	−4.5-−0.5	−1.9-0.0	−1.9-0.6	−2.7-0.3	−2.7-−0.2	−1.5-−0.6	−7.4-2.8	−2.7-0.6	−7.4-2.8
Weight *z*-score*^a^*												
*n*	1	22	29	14	16	24	17	12	3	66	72	138
Mean (SD)	−4.6 (0.0)	−1.1 (1.6)	−0.4 (1.1)	−0.5 (1.3)	−0.3 (0.2)	0.0 (0.5)	−0.2 (0.3)	−0.1 (0.4)	−0.3 (0.3)	−0.7 (1.5)	−0.2 (0.4)	−0.4 (1.1)
Median (Q1, Q3)	−4.6 (−4.6, −4.6)	−1.0 (−2.4, −0.1)	−0.4 (−1.2, 0.4)	−0.3 (−1.1, 0.4)	−0.4 (−0.5, −0.2)	−0.2 (−0.5, 0.2)	−0.2 (−0.3, 0.1)	−0.2 (−0.4, 0.0)	−0.4 (−0.5, 0.0)	−0.4 (−1.7, 0.3)	−0.2 (−0.5, 0.0)	−0.3 (−0.6, 0.0)
Min–max	−4.6-−4.6	−3.9-1.7	−2.4-1.9	−2.8-1.5	−0.6-0.3	−0.6-1.3	−0.8-0.4	−0.8-0.8	−0.5-0.0	−4.6-1.9	−0.8-1.3	−4.6-1.9
BMI *z*-score*^a^*												
*n*	1	22	29	14	16	24	14	12	3	66	69	135
Mean (SD)	−0.2 (0.0)	0.9 (0.9)	0.8 (0.7)	0.8 (0.9)	−0.1 (0.3)	0.2 (0.7)	0.1 (0.4)	0.2 (0.4)	0.1 (0.5)	0.9 (0.8)	0.1 (0.5)	0.5 (0.8)
Median (Q1, Q3)	−0.2 (−0.2, −0.2)	1.1 (0.4, 1.6)	0.7 (0.3, 1.5)	0.9 (0.0, 1.6)	−0.2 (−0.3, 0.1)	−0.0 (−0.3, 0.5)	−0.1 (−0.2, 0.3)	0.2 (−0.1, 0.4)	0.1 (−0.4, 0.6)	1.0 (0.3, 1.6)	0.0 (−0.3, 0.3)	0.3 (−0.2, 1.1)
Min-max	−0.2-−0.2	−0.6-2.0	−0.4-2.2	−0.5-1.9	−0.6-0.6	−0.5-2.5	−0.5-1.0	−0.4-0.9	−0.4-0.6	−0.6-2.2	−0.6-2.5	−0.6-2.5

Abbreviations: BMI, body mass index; CDC, Centers for Disease Control and Prevention; Q, quartile; SD, standard deviation; WHO, World Health Organization.

^*a*^z-scores calculated using CDC reference data for ages >2 years and WHO reference data for ages 0-2 years.

**Table 2:** **Medical and treatment history at baseline**.

Characteristic	<1 year (*n* = 1)	1-4 years (*n* = 22)	5-12 years (*n* = 29)	13-17 years (*n* = 14)	18-29 years (*n* = 17)	30-39 years (*n* = 24)	40-49 years (*n* = 17)	50-64 years (*n* = 12)	≥65 years (*n* = 3)	All pediatric participants (*n* = 66)	All adult participants (*n* = 73)	All participants (*n* = 139)
**Medical history, *n* (%)**												
Genu varum	1 (100.0)	14 (63.6)	23 (79.3)	11 (78.6)	13 (76.5)	21 (87.5)	14 (82.4)	10 (83.3)	2 (66.7)	49 (74.2)	60 (82.2)	109 (78.4)
Intoeing	0 (0.0)	6 (27.3)	12 (41.4)	7 (50.0)	13 (76.5)	9 (37.5)	7 (41.2)	6 (50.0)	1 (33.3)	25 (37.9)	36 (49.3)	61 (43.9)
Enthesopathy/bone spurs/ osteophytes	0 (0.0)	0 (0.0)	0 (0.0)	0 (0.0)	4 (23.5)	13 (54.2)	11 (64.7)	8 (66.7)	1 (33.3)	0 (0.0)	37 (50.7)	37 (26.6)
Osteoarthritis	0 (0.0)	1 (4.6)	0 (0.0)	0 (0.0)	0 (0.0)	10 (41.7)	11 (64.7)	9 (75.0)	1 (33.3)	1 (1.5)	31 (42.5)	32 (23.0)
Genu valgum	0 (0.0)	0 (0.0)	11 (37.9)	6 (42.9)	3 (17.7)	4 (16.7)	3 (17.7)	1 (8.3)	1 (33.3)	17 (25.8)	12 (16.4)	29 (20.9)
Tinnitus	0 (0.0)	0 (0.0)	0 (0.0)	0 (0.0)	5 (29.4)	7 (29.2)	6 (35.3)	5 (41.7)	1 (33.3)	0 (0.0)	24 (32.9)	24 (17.3)
Headache	0 (0.0)	0 (0.0)	2 (6.9)	1 (7.1)	5 (29.4)	9 (37.5)	3 (17.7)	3 (25.0)	1 (33.3)	3 (4.6)	21 (28.8)	24 (17.3)
Hearing loss	0 (0.0)	0 (0.0)	0 (0.0)	0 (0.0)	3 (17.7)	5 (20.8)	5 (29.4)	5 (41.7)	3 (100.0)	0 (0.0)	21 (28.8)	21 (15.1)
Depression	0 (0.0)	0 (0.0)	0 (0.0)	1 (7.1)	2 (11.8)	8 (33.3)	6 (35.3)	2 (16.7)	1 (33.3)	1 (1.5)	19 (26.0)	20 (14.4)
Hyperparathyroidism	0 (0.0)	0 (0.0)	1 (3.5)	0 (0.0)	4 (23.5)	4 (16.7)	4 (23.5)	4 (33.3)	1 (33.3)	1 (1.5)	17 (23.3)	18 (13.0)
Severe headache	0 (0.0)	0 (0.0)	1 (3.5)	0 (0.0)	4 (23.5)	5 (20.8)	3 (17.7)	3 (25.0)	1 (33.3)	1 (1.5)	16 (21.9)	17 (12.2)
Total number of factures, mean (SD), median (Q1, Q3), min-max*^a^*	0 (0.0)	0 (0.0)	0 (0.0)	2.8 (2.2) 2.0 (1.0, 4.0) 1.0-6.0	2.3 (1.9) 1.5 (1.0, 3.5) 1.0-5.0	3.8 (2.9) 2.5 (2.0, 7.0) 1.0-8.0	2.8 (2.9) 1.0 (1.0, 4.0) 1.0-10.0	10.4 (21.2) 3.0 (1.0, 4.0) 1.0-66.0	2.0 (0.0) 2.0 (2.0, 2.0) 2.0-2.0	2.8 (2.2) 2.0 (1.0, 4.0) 1.0-6.0	5.0 (11.5) 2.0 (1.0, 4.0) 1.0-66.0	4.7 (10.7) 2.0 (1.0, 4.0) 1.0-66.0
Non-traumatic fracture/ pseudo fracture, *n* (%)	0 (0.0)	0 (0.0)	0 (0.0)	3 (21.4)	3 (17.7)	4 (16.7)	8 (47.1)	7 (58.3)	2 (66.7)	3 (4.6)	24 (32.9)	27 (19.4)
Traumatic fracture, *n* (%)	0 (0.0)	0 (0.0)	0 (0.0)	3 (21.4)	2 (11.8)	5 (20.8)	7 (41.2)	4 (33.3)	0 (0.0)	3 (4.6)	18 (24.7)	21 (15.1)
Hypertension	0 (0.0)	0 (0.0)	0 (0.0)	0 (0.0)	1 (5.9)	2 (8.3)	6 (35.3)	4 (33.3)	1 (33.3)	0 (0.0)	14 (19.2)	14 (10.1)
Nephrocalcinosis	0 (0.0)	0 (0.0)	2 (6.9)	0 (0.0)	4 (23.5)	2 (8.3)	3 (17.7)	1 (8.3)	0 (0.0)	2 (3.0)	10 (13.7)	12 (8.6)
Spinal cord compression	0 (0.0)	0 (0.0)	0 (0.0)	0 (0.0)	0 (0.0)	4 (16.7)	4 (23.5)	2 (16.7)	1 (33.3)	0 (0.0)	11 (15.1)	11 (7.9)
Spinal surgery	0 (0.0)	0 (0.0)	0 (0.0)	0 (0.0)	0 (0.0)	2 (8.3)	2 (11.8)	0 (0.0)	1 (33.3)	0 (0.0)	5 (6.9)	5 (3.6)
**Age at XLH diagnosis,** **mean (SD), median (Q1, Q3), min-max**	0.3 (0.0) 0.3 (0.3, 0.3) 0.3-0.3	1.0 (1.0) 0.8 (0.1, 1.6) −0.1-3.0	2.2 (2.7) 1.6 (0.2, 2.6) −0.1-11.5	2.9 (4.3) 1.5 (0.2, 3.5) −0.1-13.5	1.6 (1.5) 1.1 (0.4, 2.9) −0.1-5.0	10.7 (13.3) 2.7 (1.0, 20.6) −0.1-34.5	7.7 (13.9) 1.8 (1.1, 5.9) 0.4-46.8	11.6 (21.2) 3.3 (1.5, 5.3) −0.1-64.4	55.4 (18.9) 65.7 (33.6, 66.8) 33.6-66.8	1.9 (2.8) 1.1 (0.2, 2.5) −0.1-13.5	9.9 (16.8) 2.0 (1.0, 6.6) 0.0-66.80	6.1 (12.9) 1.6 (0.4, 3.8) −0.1-66.8
**Treatment history, *n* (%)**												
Pi and/or D, ever	0 (0.0)	17 (77.3)	26 (89.7)	14 (100.0)	17 (100.0)	19 (79.2)	14 (82.4)	11 (91.7)	2 (66.7)	57 (86.4)	63 (86.3)	120 (86.3)
Pediatric	0 (0.0)	17 (77.3)	26 (89.7)	14 (100.0)	17 (100.0)	19 (79.2)	13 (76.5)	8 (66.7)	0 (0.0)	57 (86.4)	57 (78.1)	114 (82.0)
Adults	N/A	N/A	N/A	N/A	12 (70.6)	13 (54.2)	11 (64.7)	10 (83.3)	2 (66.7)	N/A	48 (65.8)	48 (34.5)
Pi and/or D, years of adult treatment, *n*, mean (SD), median (Q1, Q3), min-max	N/A	N/A	N/A	N/A	12 2.8 (3.4) 1.5 (1.0, 3.5) 0.0-11.0	12 7.7 (7.7) 3.5 (1.0, 15.0) 0.0-20.0	11 17.5 (11.5) 20.0 (5.0, 22.0) 0.0-38.0	10 10.0 (9.9) 6.5 (4.0, 15.0) 0.0-32.0	2 8.5 (12.0) 8.5 (0.0, 17.0) 0.0-17.0	N/A	47 9.3 (9.8) 5.0 (1.0, 17.0) 0.0, 38.0	47 9.3 (9.8) 5.0 (1.0, 17.0) 0.0-38.0
**Treatment at baseline, *n* (%)**												
Pi and/or D	0 (0.0)	17 (77.3)	15 (51.7)	8 (57.1)	8 (47.1)	7 (29.2)	8 (47.1)	7 (58.3)	1 (33.3)	40 (60.6)	31 (42.5)	71 (51.1)
Any pain medication	0 (0.0)	1 (4.6)	4 (13.8)	1 (7.1)	9 (52.9)	11 (45.8)	12 (70.6)	8 (66.7)	3 (100.0)	6 (9.1)	43 (58.9)	49 (35.3)
Any opioid medication	0 (0.0)	0 (0.0)	0 (0.0)	0 (0.0)	0 (0.0)	1 (4.2)	4 (23.5)	3 (25.0)	0 (0.0)	0 (0.0)	8 (11.0)	8 (5.8)

Abbreviation: SD, standard deviation.

^*a*^Total number of fractures is the sum of non-traumatic/ pseudofractures and traumatic factures.

### Use of pi/D treatment and pain medication at baseline

At baseline, most participants reported prior use of Pi/D (120/139 participants [86.3%]) (Table 2) and 71/139 participants (51.1%) reported current treatment with Pi/D. In addition, 49/139 participants (35.3%) reported currently using pain medication and 8/139 participants (5.8%) reported current opioid use; all these latter group of participants were aged ≥ 30 years (Table 2).

### Burosumab treatment duration

Median (Q1, Q3) time between baseline and Year 1 visit was 12.4 months (11.7, 14.4), with a median (Q1, Q3) burosumab treatment duration of 9.6 months (5.7, 11.6). Median (Q1, Q3) time between baseline and the Year 3 visit was 36.2 months (35.7, 36.8), with a median (Q1, Q3) burosumab treatment duration of 32.8 months (29.4, 35.4). Median (Q1, Q3) time since most recent dose at the Year 1 visit was 5.0 days (2.0, 11.0) for children and 14.0 days (7.0, 26.0) for adults. Median (Q1, Q3) time since most recent dose at the Year 3 visit was 9.5 days (5.0, 13.0) for children and 13.5 days (6.0, 25.0) for adults.

### Biochemical parameters

#### Serum phosphate concentration

At baseline, the mean (SD) serum phosphate concentration *z*-score for the overall cohort was −2.8 (0.8), with the highest mean (SD) *z*-score in those aged 50-64 years (−2.3 [0.4]) and the lowest mean (SD) *z*-score in those aged 1-4 years (−3.7 [0.7]) (Table 3). Following burosumab treatment, mean (SD) serum phosphate *z*-scores improved significantly at Year 1 and remained stable at Year 3 (change from baseline: 1.4 [1.1], *P* < .0001 and 1.4 [1.1], *P* < .0001, respectively) in the overall cohort, and showed significant improvement from baseline at both Year 1 and Year 3 in both the pediatric cohort (mean change from baseline: 1.3 [0.8], *P* < .0001 and 1.4 [1.0], *P* < .0001, respectively) and adult cohort (mean change from baseline: 1.5 [1.3], *P* < .0001 and 1.4 [1.2], *P* < .0001, respectively).

**Table 3:** **Baseline biochemistry, clinical, and physical function measures and PROs**.

Measure		<1 year	1-4 years	5-12 years	13-17 years	18-29 years	30-39 years	40-49 years	50-64 years	≥65 years	All pediatric participants	All adult participants	All participants
Serum phosphate (z-scores)*^a^*	*n*	1	21	27	12	16	24	17	12	3	61	72	133
	Mean (SD) Median (Q1, Q3) Min-max	−3.3 (0.0) −3.3 (−3.3, −3.3) −3.3-−3.3	−3.7 (0.7) −3.7 (−4.1, −3.5) −4.8-−1.8	−2.9 (0.7) −3.0 (−3.4, −2.6) −4.4-−1.3	−2.6 (0.5) −2.6 (−2.9, −2.2) −3.4-−1.9	−2.6 (0.9) −2.8 (−3.1, −2.1) −4.2-−0.6	−2.6 (0.6) −2.5 (−2.9, −2.2) −4.0-−1.6	−2.5 (0.8) −2.6 (−2.8, −2.0) −4.0-−1.2	−2.3 (0.4) −2.4 (−2.6, −1.9) −3.0-−1.6	−3.1 (0.6) −3.4 (−3.6, −2.4) −3.6-−2.4	2.7 (0.6) 2.6 (2.4, 3.0) 1.6- 4.5	2.1 (0.4) 2.1 (1.9, 2.4) 1.3-3.1	−2.8 (0.8) −2.8 (−3.4, −2.3) −4.8-−0.6
Serum alkaline phosphatase (z-scores)*^b,c^*	*n*	1	19	26	11	—	—	—	—	—	57	—	57
	Mean (SD) Median (Q1, Q3) Min-max	5.6 (0.0) 5.6 (5.6, 5.6) 5.6-5.6	3.5 (2.8) 2.6 (1.8, 4.7) 0.8-11.8	3.1 (2.1) 2.7 (1.8, 4.8) 0.2-7.0	2.1 (2.5) 1.2 (0.3, 3.4) −0.3-7.4	N/A	N/A	N/A	N/A	N/A	3.1 (2.4) 2.6 (1.7, 4.7) −0.3-11.8	N/A	3.1 (2.4) 2.6 (1.7, 4.7) −0.3-11.8
Serum iPTH (pg/mL)*^d^*	*n*	1	21	27	12	14	24	17	12	3	61	70	131
	Mean (SD) Median (Q1, Q3) Min-max	47.5 (0.0) 47.5 (47.5, 47.5) 47.5-47.5	51.2 (29.5) 40.7 (31.1, 65.9) 6.6-120.6	60.9 (28.7) 51.1 (45.0, 85.0) 16.7-152.3	67.8 (38.7) 55.3 (45.2, 91.9) 21.6-151.1	77.5 (27.1) 75.4 (53.3, 100.9) 39.1-126.3	74.8 (28.7) 67.6 (52.1, 84.2) 43.1-153.9	80.3 (30.3) 75.5 (62.8, 94.7) 40.8-139.2	98.2 (88.8) 75.8 (65.2, 85.6) 44.5-376.2	116.5 (53.3) 122.9 (60.3, 166.3) 60.3-166.3	58.7 (31.0) 50.3 (40.0, 70.8) 6.6-152.3	82.5 (45.7) 74.5 (57.9, 94.7) 39.1-376.2	71.4 (41.1) 63.5 (47.5, 85.3) 6.6-376.2
Serum 1,25(OH)_2_D (pg/mL)*^e^*	*n*	1	17	27	12	14	23	12	11	3	57	63	120
	Mean (SD) Median (Q1, Q3) Min-max	67.1 (0.0) 67.1 (67.1, 67.1) 67.1-67.1	58.7 (16.7) 54.9 (47.7, 66.6) 34.0-91.2	43.5 (20.7) 45.1 (31.8, 56.0) 10.0-82.3	42.3 (13.2) 41.9 (37.2, 53.4) 14.4-61.1	46.8 (18.1) 42.3 (36.6, 51.5) 23.0-95.1	39.4 (12.9) 37.6 (33.0, 42.9) 20.1-76.4	38.7 (19.1) 37.9 (28.4, 47.6) 10.0-73.7	40.1 (17.1) 41.1 (29.2, 44.9) 17.6-83.4	57.2 (28.2) 55.6 (29.8, 86.1) 29.8-86.1	48.2 (19.2) 48.5 (36.6, 61.1) 10.0-91.2	41.9 (16.9) 39.1 (30.6, 50.3) 10.0-95.1	44.9 (18.3) 41.5 (33.4, 55.2) 10.0-95.1
Serum creatinine (mg/dL)	*n*	1	21	27	12	16	24	17	12	3	61	72	133
	Mean (SD) Median (Q1, Q3) Min-max	0.3 (0.0) 0.30 (0.3, 0.3) 0.3-0.3	0.2 (0.1) 0.2 (0.2, 0.3) 0.2-0.3	0.4 (0.1) 0.4 (0.3, 0.4) 0.2-0.6	0.5 (0.1) 0.5 (0.4, 0.6) 0.3-0.6	0.6 (0.2) 0.6 (0.5, 0.7) 0.4-1.0	0.6 (0.2) 0.6 (0.5, 0.6) 0.4-1.2	0.7 (0.2) 0.6 (0.6, 0.8) 0.4-1.3	0.8 (0.4) 0.7 (0.6, 1.0) 0.4-1.8	0.7 (0.2) 0.7 (0.5, 0.9) 0.5-0.9	0.4 (0.1) 0.3 (0.3, 0.4) 0.2-0.6	0.67 (0.23) 0.60 (0.50, 0.75) 0.40-1.80	0.5 (0.3) 0.5 (0.4, 0.6) 0.2-1.8
RSS score*^b^*	*n*	1	20	25	7	—	—	—	—	—	53	—	53
	Mean (SD) Median (Q1, Q3) Min-max	2.5 (0.0) 2.5 (2.5, 2.5) 2.5-2.5	3.6 (2.0) 3.0 (2.0, 5.0) 1.0-8.0	1.9 (0.9) 2.0 (1.5, 2.5) 0.5-4.0	1.1 (1.4) 0.5 (0.0, 2.0) 0.0-4.0	N/A	N/A	N/A	N/A	N/A	2.4 (1.7) 2.0 (1.5, 3.0) 0.0-8.0	N/A	2.4 (1.7) 2.0 (1.5, 3.0) 0.0-8.0
TUG (seconds)*^f^*	*n*	—	—	—	—	14	22	13	11	2	—	62	62
	Mean (SD) Median (Q1, Q3) Min-max	N/A	N/A	N/A	N/A	8.2 (1.4) 8.5 (6.9, 9.5) 6.0-10.2	9.4 (1.8) 9.5 (8.1, 10.7) 6.6-13.7	9.2 (2.1) 9.3 (8.3, 10.3) 5.0-13.5	16.3 (12.0) 12.3 (10.0, 16.3) 8.4-50.6	18.0 (6.0) 18.0 (13.8, 22.2) 13.8-22.2	N/A	10.6 (6.1) 9.4 (8.2, 10.9) 5.0-50.6	10.6 (6.1) 9.4 (8.2, 10.9) 5.0-50.6
WOMAC pain score*^f^*	*n*	—	—	—	—	17	24	17	12	3	—	73	73
	Mean (SD) Median (Q1, Q3) Min-max	N/A	N/A	N/A	N/A	28.8 (19.7) 25.0 (15.0, 45.0) 0.0-60.0	37.9 (20.2) 37.5 (20.0, 55.0) 5.0-75.0	38.2 (19.7) 35.0 (25.0, 50.0) 10.0-70.0	54.6 (25.0) 52.5 (40.0, 72.5) 15.0-100.0	35.0 (15.0) 35.0 (20.0, 50.0) 20.0-50.0	N/A	38.5 (21.7) 40.0 (20.0, 55.0) 0.0-100.0	38.5 (21.7) 40.0 (20.0, 55.0) 0.0-100.0
WOMAC stiffness score*^c,f^*	*n*	—	—	—	—	17	24	17	12	3	—	73	73
	Mean (SD) Median (Q1, Q3) Min-max	N/A	N/A	N/A	N/A	50.0 (22.1) 50.0 (25.0, 75.0) 25.0-87.5	51.0 (22.1) 50.0 (37.5, 62.5) 0.0-100.0	52.9 (21.0) 50.0 (37.5, 62.5) 12.5-100.0	60.4 (29.1) 56.3 (43.8, 87.5) 12.5-100.0	45.8 (31.5) 50.0 (12.5, 75.0) 12.5-75.0	N/A	52.6 (23.1) 50.0 (37.5, 62.5) 0.0-100.0	52.6 (23.1) 50.0 (37.5, 62.5) 0.0-100.0
WOMAC PF score*^f^*	*n*	—	—	—	—	17	24	17	12	3	—	73	73
	Mean (SD) Median (Q1, Q3, Min-max	N/A	N/A	N/A	N/A	23.4 (20.1) 16.2 (4.4, 33.8) 0.0-60.3	32.6 (23.1) 36.8 (8.1, 52.9) 0.0-70.6	36.5 (19.5) 41.2 (23.5, 51.5) 0.0-60.3	52.1 (27.8) 55.9 (30.2, 67.7) 10.3-100.0	42.2 (23.0) 45.6 (17.7, 63.2) 17.7-63.2	N/A	35.0 (23.7) 36.8 (14.7, 52.9) 0.0-100.0	35.0 (23.7) 36.8 (14.7, 52.9) 0.0-100.0
PROMIS PF score*^f^*	*n*	—	—	—	—	17	24	17	12	3	—	73	73
	Mean (SD) Median (Q1, Q3) Min-max	N/A	N/A	N/A	N/A	44.4 (7.6) 42.7 (39.7, 44.4) 33.7-60.3	42.2 (9.5) 40.4 (36.6, 48.1) 24.0-60.3	38.7 (7.0) 37.9 (36.3, 42.0) 23.7-50.3	35.8 (7.9) 32.4 (30.2, 41.3) 27.3-51.4	36.3 (4.6) 35.9 (31.9, 41.0) 31.9-41.0	N/A	40.6 (8.5) 39.4 (35.9, 45.9) 23.7-60.3	40.6 (8.5) 39.4 (35.9, 45.9) 23.7-60.3
PROMIS fatigue score*^g^*	*n*	—	—	25	14	—	—	—	—	—	39	—	39
	Mean (SD) Median (Q1, Q3) Min-max	N/A	N/A	45.0 (10.6) 45.2 (35.2, 49.1) 32.4-72.3	47.3 (10.2) 50.2 (37.8, 55.3) 32.4-60.9	N/A	N/A	N/A	N/A	N/A	45.8 (10.4) 45.4 (36.5, 54.4) 32.4-72.3	N/A	45.8 (10.4) 45.4 (36.5, 54.4) 32.4-72.3
PROMIS pain interference score*^g^*	*n*	—	—	25	14	—	—	—	—	—	39	—	39
	Mean (SD) Median (Q1, Q3) Min-max	N/A	N/A	49.4 (9.1) 49.5 (39.9, 54.7) 36.8-67.6	51.1 (8.3) 53.9 (45.5, 57.1) 36.8-61.9	N/A	N/A	N/A	N/A	N/A	50.0 (8.8) 50.3 (41.4, 57.1) 36.8-67.6	N/A	50.0 (8.8) 50.3 (41.4, 57.1) 36.8-67.6
PROMIS PF mobility score*^g^*	*n*	—	—	25	14	—	—	—	—	—	39	—	39
	Mean (SD) Median (Q1, Q3) Min-max	N/A	N/A	48.5 (8.5) 48.0 (39.6, 57.2) 35.1-59.1	43.9 (7.4) 44.6 (38.5, 50.7) 33.9-53.0	N/A	N/A	N/A	N/A	N/A	46.9 (8.3) 48.0 (39.4, 53.0) 33.9-59.1	N/A	46.9 (8.3) 48.0 (39.4, 53.0) 33.9-59.1

Abbreviations: 1,25(OH)_2_D, 1,25-dihydroxyvitamin D; iPTH, intact parathyroid hormone; N/A, not applicable; PF, physical function; PRO, patient-reported outcomes; PROMIS, Patient-Reported Outcomes Measurement Information System; Q, quartile; RSS, Rickets Severity Score; SD, standard deviation; TUG, Timed Up and Go; WOMAC, Western Ontario and McMaster Universities Osteoarthritis Index; XLH-DMP, X-linked hypophosphatemia Disease Monitoring Program.

^*a*^Laboratory reference ranges serum phosphate, <1 year: 4.2-8.1 mg/dL; 1-<5 years: 3.2-6.1 mg/dL; 5-<10 years: 3.2-6.1 mg/dL; 10-<15 years: 3.1-6.0 mg/dL; ≥15 years: 2.2-5.1 mg/dL.

^*b*^Laboratory reference ranges serum alkaline phosphatase, females: ≤ 1 month: 48-406 U/L; < 1 year: 124-341 U/L; 1-<4 years: 108-317 U/L; 4-<7 years: 96-297 U/L; 7-<10 years: 69-325 U/L; 10-<15 years: 51-300 U/L; 15-<18 years: 31-110 U/L; 18-<50 years: 31-106 U/L; 50-<80 years: 35-123 U/L; 80-<90 years: 35-135 U/L; ≥90 years: 35-140 U/L. Males: ≤1 month: 75-316 U/L; <1 year: 82-383 U/L; 1-<4 years: 104-345 U/L; 4-<7 years: 93-309 U/L; 7-<10 years: 86-315 U/L; 10-<15 years: 95-385 U/L; 15-<18 years: 50-250 U/L; 18-<50 years: 31-129 U/L; 50-<60 years: 35-131 U/L; 60-<70 years: 35-125 U/L; 70-<80 years: 35-130 U/L; ≥80 years: 35-125 U/L.

^*c*^Serum alkaline phosphatase data and RSS score not collected for adults per XLH-DMP protocol.

^*d*^Laboratory reference ranges serum iPTH, 18.4-80.1 pg/mL.

^*e*^Laboratory reference ranges serum 1,25(OH)_2_D, 18.0-72.2 pg/mL.

^*f*^TUG, WOMAC pain score, WOMAC stiffness score, WOMAC PF score, and PROMIS PF score not assessed in children per XLH-DMP protocol.

^*g*^PROMIS fatigue score, PROMIS pain interference score, and PROMIS mobility score were assessed in the 5-12 and 13-17 years age groups only per XLH-DMP protocol.

**Figure 2:**
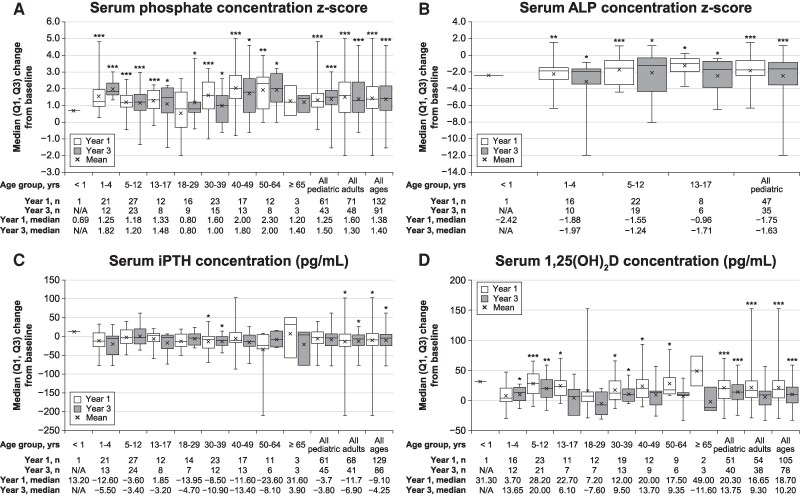
Change from baseline in biochemical parameters at Year 1 and Year 3; (A) serum phosphate *z*-scores; (B) serum alkaline phosphatase (ALP) *z*-scores; (C) serum iPTH concentrations; (D) serum 1,25(OH)_2_D concentrations. *P*-values apply to the mean change from baseline. *P < .05; **P < .001; ***P < .0001. Abbreviations: 1,25(OH)_2_D, 1,25-dihydroxyvitamin D; ALP, alkaline phosphatase; iPTH, intact parathyroid hormone; yrs, years.

Statistically significant improvements were seen in 6 of the 9 age groups at Year 1 and 7 of the 9 age groups at Year 3 (Fig. 2A) (25). In the overall cohort, 45/133 participants (33.8%) had a serum phosphate concentration within reference range at baseline; following burosumab treatment, 113/136 participants (83.1%) at Year 1 and 82/93 participants (88.2%) at Year 3 were within age-appropriate reference ranges (Table S3) (26).

#### Serum alkaline phosphatase concentration

At baseline, the mean (SD) serum alkaline phosphatase *z*-score for the overall pediatric cohort was 3.1 (2.4) and ranged from 2.1 (2.5) in those aged 13-17 years to 5.6 (0.0) in the participant aged < 1 year (Table 3). Mean (SD) serum alkaline phosphatase *z*-scores decreased significantly following burosumab treatment at both Year 1 and Year 3 (change from baseline: −1.9 [1.7], *P* < .0001 and −2.5 [2.6], *P* < .0001, respectively) in the overall cohort, with reductions in all pediatric age categories at Year 1, and greater reductions observed at Year 3. Reductions from baseline were statistically significant for all age groups with more than one participant included (Fig. 2B) (25). At baseline, 57/91 (62.6%) were above the upper limit of normal range (ULN). This decreased to 38/66 participants (57.6%) and 14/45 participants (31.1%) above the ULN at Year 1 and Year 3, respectively.

#### Serum intact parathyroid hormone concentration

At baseline, the mean (SD) serum iPTH concentration for the overall cohort was 71.4 (41.1) pg/mL and within the reference range. However, mean serum iPTH concentration exceeded the reference range in older adults (aged 40-49 years, 50-64 years, and ≥ 65 years) (Table 3). Following burosumab treatment, mean (SD) serum iPTH concentration for the overall cohort decreased significantly from baseline at Year 1 (change from baseline: −9.5 [32.4] pg/mL, *P* = .001) and Year 3 (−9.9 [26.9] pg/mL, *P* = .001). Non-significant decreases from baseline were seen at both Year 1 (−5.5 [24.9] pg/mL, *P* = .092) and Year 3 in the pediatric cohort (−8.1 [29.7] pg/mL, *P* = .076), and significant decreases were seen at both Year 1 (−13.0 [37.7] pg/mL, *P* = .006) and Year 3 in the adult cohort (−11.8 [23.8] pg/mL, *P* = .003). Statistically significant decreases were seen in the 30-39 years group at both Year 1 and 3 (Fig. 2C).

In the overall cohort, 78/131 participants (59.5%) had a serum iPTH concentration within the normal reference range at baseline; following burosumab treatment 102/136 participants (75.0%) at Year 1 and 75/89 participants (84.3%) at Year 3 were within the normal reference range (Table S4) (26). At baseline, 3/38 participants (7.9%) with an iPTH concentration above the ULN also had hypercalcemia. At Year 1, 3/30 participants (10.0%) with an iPTH concentration above ULN had hypercalcemia, while at Year 3, no participants (0/13, 0.0%) with an iPTH concentration above ULN had hypercalcemia.

#### Serum 1,25-dihydroxyvitamin D concentration

At baseline, mean (SD) serum 1,25(OH)_2_D concentration was 44.9 (18.3) pg/mL in the overall cohort (Table 3) (25). Following burosumab treatment, mean (SD) serum 1,25(OH)_2_D concentration increased significantly at Year 1 and Year 3 (change from baseline: 21.3 [26.4], *P* < .0001 and 10.1 [20.6], *P* < .0001, respectively) in the overall cohort, significant increases were seen at Year 1 (21.1 [22.5], *P* < .0001) and Year 3 (14.2 [20.3], *P* < .0001) in the pediatric cohort, and in the adult cohort a significant increase was seen at Year 1 (21.5 [29.9], *P* < .0001) and a non-significant increase was seen at Year 3 (5.9 [20.3], *P* = .084).

Statistically significant increases were seen in 5 of the 9 age groups, and non-significant increases in the remaining 4 age groups at Year 1 (Fig. 2D) (25). At Year 3, mean 1,25(OH)_2_D concentration increased from baseline but decreased compared to Year 1 values for most age groups, except for those aged 18-29 and ≥ 65 years for whom mean concentration decreased from baseline (Fig. 2D). Overall, increased levels compared to baseline were seen for 85 participants (81%) at Year 1 and 56 participants (72%) at Year 3. At baseline, 104/120 participants (86.7%) had a serum 1,25(OH)_2_D concentration within the normal reference range. This increased to 111/120 participants (92.5%) and 76/86 participants (88.4%) at Year 1 and Year 3, respectively (Table S5) (26).

#### Serum creatinine concentration

At baseline, mean (SD) serum creatinine concentration was 0.5 (0.3) mg/dL in the overall cohort (Table 3) (25). Following burosumab treatment, serum creatinine concentration increased in the overall cohort, with a mean (SD) change from baseline to Year 1 of 0.04 (0.09) mg/dL (*P* < .0001) and 0.10 (0.13) mg/dL (*P* < .0001) at Year 3 (Table S6) (26). At baseline, 130/133 participants (97.7%) were within the reference serum creatinine range. Following burosumab treatment, 135/136 participants (99.3%) at Year 1 and 89/92 participants (96.7%) at Year 3 were within the reference serum creatinine range.

### Rickets severity score in pediatric participants

At baseline, mean (SD) total RSS for the pediatric cohort was 2.4 (1.7). Following burosumab treatment, mean (SD) total RSS decreased significantly at Year 1 (change from baseline: −1.5 [1.5], *P* < .0001) and Year 3 (−1.5 [1.7], *P* < .0001). Improvements from baseline were observed across all age groups, with significant improvements in the 1-4 year and 5-12-year groups at both the Year 1 and Year 3 visits (Table S7) (26).

### Functional assessment in adult participants

At baseline, mean (SD) TUG time in adult participants was 10.6 (6.1) seconds (Table 3). TUG times tended to increase with advancing age, except for the 40-49-year group. A statistically significant improvement in mean (SD) TUG time was observed following burosumab treatment in the overall adult cohort at Year 1 (change from baseline: −1.2 [2.9] seconds, *P* = .003), with non-significant changes observed at Year 3 (−0.9 [2.8] seconds, *P* = .051). Statistically significant improvements were observed in the 50-64-year group at Year 1 (−3.7 [3.7], *P* = .011) and in the 30-39-year group (−0.7 [1.0], *P* = .043) at Year 3 (Table S8) (26).

### Patient-reported outcomes in adult participants

At baseline for the adult cohort, mean (SD) WOMAC pain score was 38.5 (21.7), mean (SD) WOMAC stiffness score was 52.6 (23.1), and mean (SD) WOMAC PF score was 35.0 (23.7) (Table 3) (25). There was a non-significant increase in all baseline WOMAC scores, suggesting worse health, with increasing age, except for the ≥ 65 years group, which included only 3 participants (Table 3).

**Figure 3:**
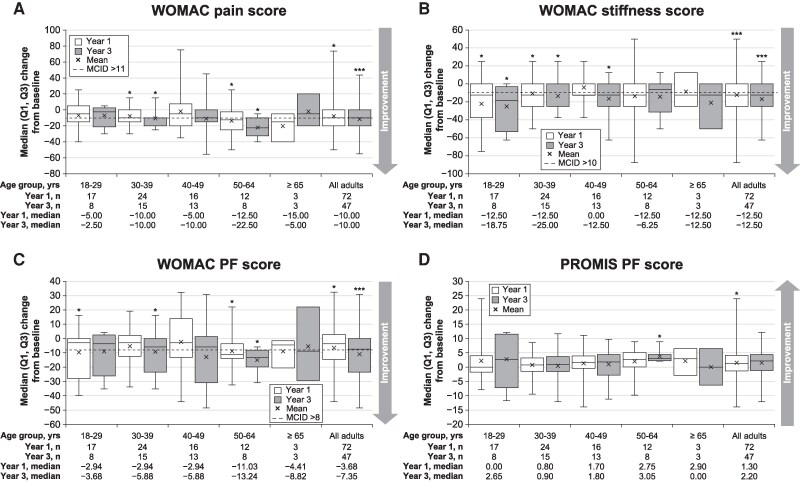
Change from baseline in PROs in adult participants at Year 1 and Year 3 following burosumab treatment; (A) WOMAC pain scores; (B) WOMAC stiffness scores; (C) WOMAC PF scores; (D) PROMIS PF scores^a^. *P*-values apply to mean change from baseline. *P < .05, ***P < .0001. ^a^Scoring is a summed metric normalized to 0-100, representing the percent of the maximum score, where 0 is the best state of health, and 100 is the worst. Abbreviations: PF, physical function; PRO, patient-reported outcomes; PROMIS, Patient-Reported Outcomes Measurement Information System; Q, quartile; WOMAC, Western Ontario and McMaster Universities Osteoarthritis Index; yrs, years.

Following burosumab treatment, improvements in mean (SD) scores were observed in all 3 WOMAC domains in the overall adult cohort at Year 1 (pain: −7.5 [18.8], *P* = .001; stiffness: −12.2 [23.6], *P* < .0001; PF: −6.3 [15.5], *P* = .001), with more pronounced improvement for PF at Year 3 (pain: −11.3 [16.9], *P* < .0001; stiffness: −16.8 [21.7], *P* < .0001; PF: −10.8 [16.6], *P* < .0001) (Fig. 3A-3C).

WOMAC changes in the age sub-groups were generally consistent with the overall cohort. Specifically, the participants aged 18-29 years showed significant improvements in stiffness, participants aged 30-39 years experienced significant improvements in pain and stiffness, and participants aged 50-64 years experienced significant improvements in pain and PF at both the Year 1 and Year 3 visits (Fig. 3A-3C).

**Figure 4:**
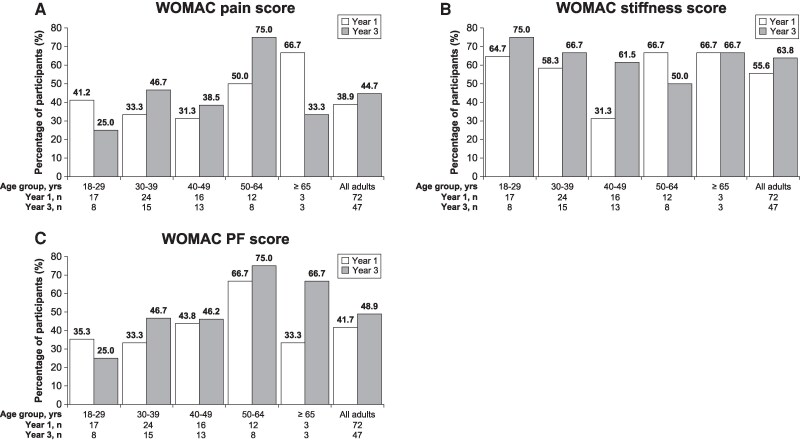
Percentage of adult participants achieving MCID in WOMAC (A) pain, (B) stiffness, and (C) PF at Year 1 and Year 3. Abbreviations: MCID, minimally clinically important difference; PF, physical function; WOMAC, Western Ontario and McMaster Universities Osteoarthritis Index; yrs, years.

Across the overall adult cohort, a higher proportion of participants achieved improvements greater than the MCID for each WOMAC domain at Year 3 compared with Year 1: pain: 21/47 (44.7%) vs 28/72 (38.9%); stiffness: 30/47 (63.8%) vs 40/72 (55.6%); PF: 23/47 (48.9%) vs 30/72 (41.7%) (Fig. 4).

At baseline, mean (SD) PROMIS PF scores worsened with increasing age, with values ranging from 44.4 (7.6) in patients aged 18-29 years to 35.8 (7.9) in those aged 50-64 years (Table S9) (26). In the overall adult cohort, mean (SD) PROMIS PF improved significantly at Year 1 (change from baseline: 1.5 (5.6), *P* = .025) with a non-significant change at Year 3 (1.6 [5.8], *P* = .070). Although not statistically significant, results in the age sub-groups were generally consistent with the overall cohort, except for the 50-64-year group in which an additional increase was observed at Year 3 (3.8 [2.3], *P* = .002) and in the ≥65-year group, where the improvement at Year 1 (2.2 [4.7], *P* = .512) was not observed at Year 3 (0.1 [6.4], *P* = .987) (Fig. 3D).

### Patient-reported outcomes in pediatric participants

**Figure 5:**
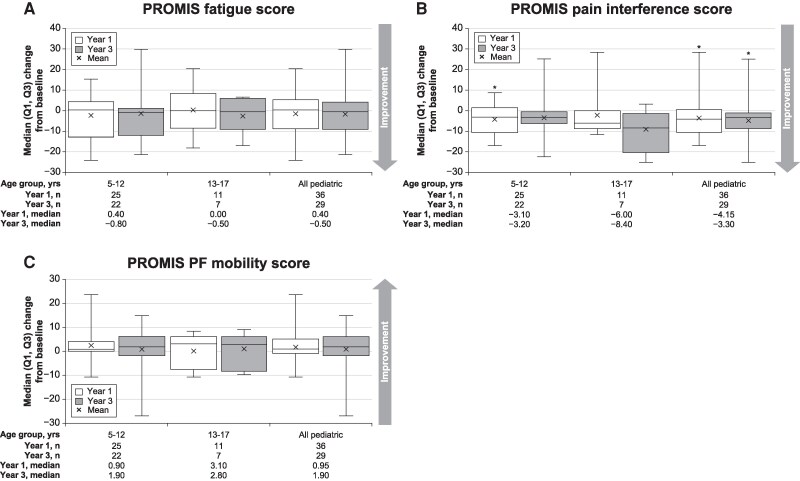
Change from baseline in PROs in pediatric participants at Year 1 and Year 3 following burosumab treatment; (A) fatigue, (B) pain interference, and (C) PF mobility. *P*-values apply to the mean change from baseline. *P < .05. Abbreviations: PF, physical function; PRO, patient-reported outcomes; PROMIS, Patient-Reported Outcomes Measurement Information System; Q, quartile; yrs, years.

At baseline for the pediatric cohort, the mean (SD) PROMIS fatigue score was 45.8 (10.4), pain interference score was 50.0 (8.8), and PF mobility score was 46.9 (8.3) (Table S10) (26). Mean (SD) PROMIS pain interference improved significantly at Year 1 (change from baseline: −3.6 [8.5], *P* = .016) and Year 3 (−4.8 [10.1], *P* = .017). Non-significant changes were observed in PROMIS fatigue at Year 1 (−1.4 [10.0], *P* = .396) and at Year 3 (−1.6 [11.7], *P* = .458) and PROMIS PF mobility at Year 1 (1.8 [6.7], *P* = .128) and at Year 3 (0.9 [8.6], *P* = .057). In the pediatric age sub-groups, non-significant improvements were observed in all 3 PROMIS domains following burosumab treatment, except for PROMIS pain interference in participants aged 5-12 years at Year 1 (Fig. 5A-5C).

### Safety

AEs of special interest were evaluated for 776 participants (347 children and 429 adults) enrolled in the XLH-DMP between June 2018 and December 2022 (enrollment cut-off: 29 December 2022).

#### Renal health

Seven non-serious treatment-related AEs related to renal health were reported in adult participants: nephrocalcinosis in 4 participants (1 case in each), proteinuria in 2 participants (1 event each), and 1 event of elevated blood creatinine in 1 participant (Table S11) (26). Three serious AEs (SAE) of acute kidney injury were also reported in 2 adult participants (2 events in one participant), but were assessed as unrelated to burosumab. No renal AEs were identified in pediatric patients. Renal laboratory parameters, including serum creatinine, estimated glomerular filtration rate (eGFR), and urine protein-to-creatinine ratio, generally remained within normal ranges.

#### Spinal stenosis

A total of 16 SAEs related to spinal stenosis were reported in 13 adult participants (Table S11) (26). One of these events (spinal pain) was assessed as related to burosumab by the Investigator and the Sponsor, while the remaining events were assessed as not related by both. Nine of these participants had a history of spinal stenosis prior to entering the XLH-DMP.

#### Pregnancy and lactation

Thirty-two participants became pregnant during the XLH-DMP, out of which 19 adults and 1 pediatric participant were exposed to burosumab prior to conception. A total of 32 pregnancies resulted in live births, 5 resulted in spontaneous abortion/miscarriage, 4 in birth defect/congenital anomaly, and 1 in ectopic pregnancy. Thirteen pregnancy-related SAEs were reported, none of which were considered related to burosumab. One infant was exposed to burosumab through breastfeeding; no AEs were reported in either the infant or the mother.

## Discussion

This analysis of 139 participants with XLH who received burosumab treatment following enrollment in the XLH-DMP reports burden of disease at baseline, and changes in biochemical parameters, clinical assessments, functional assessment, and PROs after 1 and 3 years of burosumab treatment. Outcomes are reported across a range of age categories (< 1 year to ≥65 years), including those age groups previously excluded from pivotal phase 3 clinical trials. We show that mean serum phosphate z-scores increased from baseline at Year 1 and Year 3, for the entire cohort and across age groups. Among pediatric participants, alkaline phosphatase z-scores declined significantly in Year 1, with further improvements at Year 3, for all age groups > 1 year of age. Mean RSS improved from baseline for the pediatric cohort as a whole and across age groups at Years 1 and 3. Improvements were also seen within PRO results; children with XLH showed significant decreases in pain interference while adults experienced improvements across all WOMAC domains at both Year 1 and 3. These results support and extend findings from pivotal phase 3 trials showing that burosumab treatment in participants with XLH led to reduced alkaline phosphatase and improved rickets in children, decreased stiffness in adults, and improved PROs and normalized serum phosphate in all participants (12, 13). Observed trends were generally consistent across age groups, highlighting the sustained effectiveness of burosumab treatment across the lifespan of individuals with XLH.

At baseline, many participants exhibited marked hypophosphatemia. Hypophosphatemia is an important mediator of many XLH-related complications, including rickets and poor growth in children, osteomalacia, and lower limb deformity across all ages. The observed hypophosphatemia at baseline is consistent with the effects of excess FGF23 in the absence of a prior therapeutic strategy that specifically inhibits FGF23 activity for individuals with XLH. Following burosumab treatment, mean serum phosphate concentration and *z*-scores improved significantly in most age groups at Year 1, in line with previous phase 3 studies in both children and adults (12, 13), and this improvement was maintained over the 3 years of treatment. Additionally, 83.1% and 88.2% of participants achieved age-appropriate normal serum phosphate at Year 1 and 3, respectively, vs 33.8% at baseline. These findings suggest that ongoing FGF23 neutralization resulted in sustained improvements in renal phosphate handling during burosumab treatment.

Mean 1,25(OH)_2_D concentration was within reference range at baseline and increased significantly during burosumab treatment, consistent with the findings from the pediatric phase 3 trial, and a non-significant increase from baseline was also seen in the adult phase 3 trial (12, 13), supporting effective FGF23 inhibition. According to current understanding, burosumab-mediated FGF23 neutralization increases 1,25(OH)_2_D synthesis, which enhances intestinal absorption of phosphate and calcium. Increased calcium absorption then helps to counteract the PTH-stimulatory effect of the increasing serum phosphate concentration (27). Improved serum phosphate and endogenous 1,25(OH)_2_D concentration are signature biochemical responses to burosumab that are not achieved during treatment with Pi/D due to the well-documented, persistent, excess FGF23 secretion with these oral supplements (28).

Mean alkaline phosphatase concentrations and *z*-scores decreased with burosumab treatment in all pediatric categories at Year 1, supporting the results from the phase 3 trial (12), with greater declines observed at Year 3. The decline in serum alkaline phosphatase, a key biomarker of rickets severity, reflects improvements in bone mineralization over time, providing additional support for long-term use of burosumab (13).

Mean iPTH concentration was elevated at baseline in this study population and decreased at Year 1 and Year 3 following burosumab treatment, consistent with pivotal randomized phase 3 trials in adults (13) and in a real-world analysis of adults treated with burosumab (26). Furthermore, the number of participants with iPTH above the ULN fell from 40.5% at baseline to 15.7% after 3 years of burosumab treatment, with 84.3% of participants registering an iPTH concentration within the reference range at the Year 3 timepoint. PTH is critical for phosphate homeostasis, with elevations in PTH promoting bone resorption and phosphaturia (26). Hyperparathyroidism is associated with significant patient burden, especially when parathyroid autonomy develops, which then requires either concomitant adjuvant medical therapy (ie, a calcimimetic medication) targeting PTH over-secretion or a parathyroidectomy. Therefore, the decline in iPTH with burosumab suggests an improvement in parathyroid homeostasis (29).

In terms of renal function, this analysis showed that, although there was a small increase in serum creatinine during burosumab treatment, most values in pediatric and adult participants remained within reference ranges. These findings are consistent with previous reports showing mild increases in serum creatinine in participants on burosumab but no evidence of renal dysfunction (12, 13) and support the need for continued renal function monitoring during burosumab treatment.

At baseline, older adults reported more pain and stiffness, diminished mobility, increased prevalence of musculoskeletal conditions, and higher use of pain medications, including opioids, compared with younger adults. These findings align with previous reports describing the progressive burden of XLH with increasing age and its impact on health-related quality of life (30, 31).

PRO and physical function improvements were observed at Year 1 and were maintained at Year 3, with significant gains across all 3 WOMAC domains (pain, stiffness, and PF) and sustained improvements in PROMIS PF and TUG in adults and PROMIS pain interference scores in children. All age groups showed improvements, with many participants achieving the MCID for each domain. These findings are consistent with a previous phase 3 study that also showed significant improvements in all assessed WOMAC domains (pain, stiffness, and PF), Brief Pain Inventory-Short Form (BPI-SF), and TUG after 48 weeks in adults treated with burosumab (14).

Others have also reported on the real-world effectiveness of burosumab in the treatment of XLH for pediatric or adult cohorts and consistently demonstrate increases in serum phosphate concentration and indicate improvements in PROs, such as WOMAC (32-35). However, to the best of our knowledge, no other published real-world studies of burosumab treatment have reported results for both pediatric and adult cohorts, stratified by age group, as in this study.

An Italian XLH cohort of 27 adult patients, with a similar age distribution to the adult cohort in our study, demonstrated significantly increased serum phosphate concentration levels after 24 weeks, and a subgroup of 11 patients demonstrated significantly improved WOMAC pain, stiffness, and physical function scores after 48 weeks of a similar magnitude to our study (32). A longitudinal report from the European International XLH registry followed 67 children and adolescents with a mean duration of burosumab exposure of 29.7 months (SD 25) (33). This publication, however, was focused on safety and adverse event reporting with limited biochemistries reported, and no PROs. A German cohort of 93 children and adolescents with XLH was treated with burosumab for 12 months (34). This German study demonstrated a similar magnitude of improvement in serum phosphate concentration in both children and adolescents, and increases in 1,25(OH)_2_D were also seen after 1 year of treatment. However, in our study, a higher proportion of pediatric participants had achieved normal serum phosphate concentration at Year 1 and Year 3 (59–100% depending on age group) compared to 42% at Year 1 in the German cohort. For the mean alkaline phosphatase z-score, by Year 1, there was a decrease of 1.3 (80% of patients achieved normalization) in the German cohort. Our study reported a similar decrease, although a smaller proportion of participants achieved normalization by Year 1 (42.2%) and Year 3 (68.9%). Adults in the UK who were treated with burosumab (*n* = 136, median [Q1, Q3] age 44.0 years [18, 83]) demonstrated normalization of serum phosphate concentration in 63% of patients (compared with 5% at baseline) at 6 months, 50% at 12 months, and 41% at 18 months; improvements in patient-reported pain, stiffness, and physical function (using Brief Pain Inventory—Short Form and WOMAC) at 6, 12, and 18 months were noted, similar to our findings (35).

The safety findings of interest assessed in the XLH-DMP are consistent with trends reported in previous clinical trials of burosumab, with no new safety concerns identified. There was no evidence to suggest an increased risk of nephrocalcinosis, renal failure, spinal stenosis, or spinal cord compression. In addition, no safety signals related to pregnancy, lactation, or neonatal outcomes were observed. It is important to note, however, that the current international guidelines for the management of XLH in adults do not provide recommendations regarding use of burosumab during pregnancy and lactation due to the lack of sufficient safety data (10). Nonetheless, the real-world data presented here support the continued favorable benefit–risk profile of burosumab in both pediatric and adult patients with XLH (12, 13).

This analysis has several limitations, including sample size, geographical distribution, and the real-world design of the DMP. Although the study included 139 participants with XLH across a broad age range, there were few participants in the youngest (<1 year) and oldest (≥65 years) cohorts, limiting interpretation for these groups. In addition, most participants (89/139; 64%) were from the United States, which may impact global generalizability due to variations in healthcare delivery. Additionally, the real-world setting introduces variability in the timing of burosumab initiation, treatment duration, and the timing of dosing in relation to visits for biochemical and other assessments, and more missing data compared with clinical trials. These factors could influence outcomes at the Year 1 and Year 3 visits. As this was an exploratory observational study, there were no pre-specified hypotheses, and as such, an adjustment for multiplicity was not applied to any analysis; *P*-values were nominal only. Finally, it is recognized that the establishment of an MCID for rickets severity in pediatric XLH would facilitate data interpretation in the context of real-world, treatment effectiveness trials such as this.

In conclusion, in this real-world analysis, burosumab treatment over 3 years was associated with improvements in key biochemical outcomes and PROs in participants with XLH aged < 1 to ≥ 65 years. The ongoing XLH-DMP aims to characterize the long-term effectiveness of burosumab treatment over a full decade.

## Data Availability

The data that support the findings of this study are available from Kyowa Kirin, upon reasonable request.

## Clinical trial information

ClinicalTrials.gov no. NCT03651505 (registered August 24, 2018).

## Abbreviations

1,25(OH)_2_D: 1,25-dihydroxyvitamin D
AE: adverse events
BMI: body mass index
D: activated vitamin D
DMP: Disease Monitoring Program
FGF23: fibroblast growth factor 23
iPTH: intact parathyroid hormone
IQR: interquartile range
MCID: minimal clinically important difference
N/A: not-applicable
PF: physical function
*PHEX*: phosphate-regulating endopeptidase homolog X-linked
Pi: phosphate salts
PRO: patient-reported outcome
PROMIS: Patient-Reported Outcomes Measurement Information System
PTH: parathyroid hormone
Q: quartile
RSS: Rickets Severity Score
SAE: serious adverse event
SC: subcutaneously
SD: standard deviation
TUG: Timed Up and Go
WOMAC: Western Ontario and McMaster Universities Osteoarthritis Index
XLH: X-linked hypophosphatemia
Y: Year
Yr: Years
